# Sinusoidal Fitting Decomposition for Instantaneous Characteristic Representation of Multi-Componential Signal

**DOI:** 10.3390/s24217032

**Published:** 2024-10-31

**Authors:** Donghu Nie, Xin Su, Gang Qiao

**Affiliations:** 1 National Key Laboratory of Underwater Acoustic Technology, Harbin Engineering University, Harbin 150001, China; suxin_@hrbeu.edu.cn (X.S.); qiaogang@hrbeu.edu.cn (G.Q.); 2 Key Laboratory of Marine Information Acquisition and Security (Harbin Engineering University), Ministry of Industry and Information Technology, Harbin 150001, China; 3 College of Underwater Acoustic Engineering, Harbin Engineering University, Harbin 150001, China; 4 Sanya Nanhai Innovation and Development Base of Harbin Engineering University, Sanya 572024, China

**Keywords:** multi-component signal decomposition, instantaneous frequency, instantaneous phase, time-frequency analysis, non-stationary signal processing

## Abstract

The research on how to effectively extract the instantaneous characteristic components of non-stationary signals continues to be both a research hotspot and a very challenging topic. In this paper, a new method of multi-component decomposition is proposed to decompose a signal into finite mono-component signals and extract their Instantaneous Amplitude (IA), Instantaneous Phase (IP), and Instantaneous Frequency (IF), which is called Sinusoidal Fitting Decomposition (SFD). The proposed method can ensure that the IA extracted from the given signal must be positive, the IP is monotonically increasing, and the signal synthesized by both IA and IP must be mono-componential and smooth. It transforms the decomposition process into a synthesis iterative process and does not rely on any dictionary or basis function space or carry out the sifting operation. In addition, the proposed method can describe the instantaneous-frequency-amplitude characteristics of the signal very well on the time-frequency plane. The results of numerical simulation and the qualitative analysis of the amount of calculation show that the proposed method is effective.

## 1. Introduction

Multi-component non-stationary signals are the most common signals in the real world and widely exist in sonar, radar, and other applications. The most efficient way to analyze and process a non-stationary signal is to exact its Instantaneous Frequency (IF), Instantaneous Phase (IP), and Instantaneous Amplitude (IA). In the past few decades, most of the methods [1,2,3,4] are for stationary signals, but the processing results are often unsatisfactory. The similarity of these methods is that they must calculate the inner product of the signal and some kernel functions or basis functions over a whole-time interval, to obtain the time-frequency energy concentration in the time-frequency plane and estimate the IF. Therefore, they belong to global transform methods and have to comply with the Heisenberg uncertainty principle [5], which makes it impossible to obtain a good resolution in both time and frequency domains simultaneously. Due to the limitations of stationary time-frequency analysis, people have been trying to find other methods to extract the instantaneous characteristics of signals, especially the IF.

In the 1930s, Carson and Fry [6] first proposed the concept of IF, and then Van der Pol [7] gave a specific definition that IF is the derivative of the phase of the Frequency Modulation (FM) signal, which is one of the most commonly accepted definitions thus far and laid the theoretical foundation of non-stationary signal analysis. Gabor [8] introduced Hilbert Transform (HT) to obtain the unique Analytic Signal (AS) representation of the real-valued signal, so an Amplitude Modulation and Frequency Modulation (AM-FM) signal can be represented with a unique pair of amplitude and phase. Based on the previous research, Ville [9] used the derivative of the AS phase based on HT to define the IF, which was the beginning of the practical application of the IF. In addition, Bedrosian [10] proposed a very useful solution of the product theorem, where the core idea of this theory is that HT can be used to obtain an effective AS only when the frequency spectra of amplitude and phase of an AM-FM signal are fully separated, which has been proved theoretically. According to the above theory from Bedrosian and the definition of IF from Ville, the signal must be mono-componential, otherwise the obtained IF is meaningless. Thus, Boashash [11] established the model of a non-stationary, multi-component signal that is expressed as the weighted sum of finite mono-component (MC) signals, each one with its own IF. Qian [12,13] has given an accurate mathematical definition of being a mono-component, and has conducted considerable work in building a family of MC classes [14].

The questions of how to construct MCs and how to decompose a signal into MC signals have become a research hotspot of non-stationary signal analysis. Sharpley [15] pointed out that two things must be carried out in the multi-component decomposition: firstly, an appropriate method must be selected to decompose the signal into MCs, and then the amplitude and phase of each component are determined by HT. At present, most of the multi-component decomposition methods mainly adopt the following three technical routes:

The first type of method is to extract all MC signals from a given signal through iterative sifting. The Empirical Mode Decomposition (EMD) proposed by Huang [16] is completely data-adaptive and does not rely on any basis function dictionary (such as the Fourier transform basis function, or mother wavelet of wavelet transform), so it is widely used due to its simplicity and effectiveness [17]. In order to solve the problems of mode aliasing and completeness, some improved methods have been proposed such as the Ensemble Empirical Mode Decomposition (EEMD) in [18]. Dragomiretskiy and Zosso [19] proposed the Variational Mode Decomposition (VMD) method for decomposing a signal into an ensemble of band-limited IMFs. VMD uses the principle of Wiener filter denoising and the optimization iterative method to sift the component modes from the signal and focus the component signal near the center frequency of each mode. Compared with EMD and those improved EMD methods, VMD can better improve the mode mixing and endpoint effect, and can obtain better time-frequency properties [20]. But VMD cannot extract the IFs from signals directly and usually needs to use the Hilbert transformation as post-processing to obtain the instantaneous characteristics [21,22,23].

The second type of method is to construct a redundant MC dictionary, and then use an optimization method to decompose the signal into a weighted sum of the MC atoms in the dictionary through iteration. Qian [24,25] proposed a multi-component decomposition method called Adaptive Fourier Decomposition (AFD); the method constructs an atom dictionary by a rational and orthogonal system or Takenaka-Malmquist system, and then calculates the optimal linear combination of processed signals in the dictionary through iteration and the greedy algorithm. The algorithm is somewhat similar to the matching pursuit algorithm, so it also faces the problem of large amounts of computation, but its advantages are also obvious. The atomic functions in the dictionary are mono-componential, which ensures that the decomposition result is mono-componential [14]. Hou [26,27] proposed several decomposition methods for sparse multi-component signals. The core idea is to build a highly redundant dictionary, and then sparsely decompose the signal over the dictionary. In this way, the decomposition becomes an optimization problem for solving nonlinear problems. Hou’s method is a partially variational approach to the traditional EMD [28], which to a certain extent, also belongs to the first technical route, but they use a sifting method different from EMD.

The third type of method is the sparse reconstruction based on a time-frequency transformation. Daubechies [29] proposed an EMD-like tool called Synchro-Squeezing Transform (SST) and introduced a class of Intrinsic Mode Type (IMT) functions. This method combines the idea of wavelet analysis and time-frequency energy reassignment to squeeze the Continuous Wavelet Transform (CWT) over regions where the phase transform is constant, then to obtain a sharper time-frequency representation. Gilles [30] proposed an approach called Empirical Wavelet Transform (EWT), which extracts the appropriate wavelet filter band from the signal and then decomposes the signal into adaptive sub-bands by reconstruction. This method relies heavily on the robustness of peak detection, spectrum segmentation, and filter band construction.

In addition to the above methods, Hou [26,27] and Huang [5] also proposed different methods to extract IA, IF, and IP directly. In Hou’s methods, according to whether the data are periodic, the signal is decomposed into Fourier basis or some basis, and Newton–Raphson-based or ODE-based, EMD methods are used to solve the nonlinear problem, respectively, after which the IP and IA can be obtained. The MC signal is extracted from the signal by a nonlinear sifting program, and the discrete second-order difference method is used to calculate the IF. The variational methods provide similar results as EMD. In [5], an improved method called the Normalized Hilbert Transform (NHT) is proposed, which uses the recursive method to normalize the Intrinsic Mode Function (IMF) component itself with the envelope of the absolute value of IMF, transforms IMF into FM function of unit amplitude, and then the IP and IF can be solved by the complex analytic function of unit amplitude. The IA can be obtained by dividing the original IMF by the FM function of unit amplitude. Huang’s method carries out the second iterative operation over EMD to obtain the instantaneous characteristics but increases the amount of computation. In addition, with the development of deep learning (DL) technology, some researchers have used it for signa decomposition [31] and time-frequency analysis [32], trying to break through the limitations of traditional methods.

In this paper, a new multi-component decomposition method called Sinusoidal Fitting Decomposition (SFD) is proposed, which is completely different from the above methods. The innovation lies in the new method of extracting IA, IP, and IF directly from a given signal, which transforms the decomposition process into a synthesis iterative process. Compared with the above methods, the proposed method for extracting IP and IA is simpler and does not need iterative sifting or solving an optimization problem. This method only uses the local maximums of the signal as the anchor points and realizes the fitting of IA and IP through cubic Hermite polynomial interpolation. The obtained IP and IA curves are first-order smooth. Then, the MC signal in the form of AM-FM is synthesized by the IA and IP pair, both of which can meet the conditions of a MC signal automatically. Subtracting this component from the original signal, just like EMD, repeats the above process in the residual signal to identify all MCs. The IF can then be calculated directly through the derivative of IP without an HT.

The decomposition process of the proposed method is similar to VMD but does not need a sifting operation or a dictionary. The calculation is simpler, easier to understand, and is not limited by the narrowband hypothesis. The instantaneous-frequency-amplitude spectrum, which is called Sinusoidal Fitting Spectrum (SFS) in this paper, can be clearly expressed on the time-frequency plane by using our method, which is beneficial to the analysis of non-stationary time-varying signals.

The rest of this paper is organized as follows: Section 2 briefly reviews the concept of IF and MC. Section 3 introduces the SFD method in detail. Section 4 shows the simulation results and the real acoustic signal processing results. Finally, we give our conclusions in Section 5.

## 2. Theoretical Backgrounds

In order to illustrate the proposed algorithm, some relevant definitions are first reviewed.

### 2.1. Review of IF Definition

**Definition 1.**  *Let $\phi \left(t\right)$ be the phase of the harmonic signal, the signal can be expressed in the AM-FM form*

(1) $$x\left(t\right)=a\left(t\right)cos\left(\phi \left(t\right)\right)$$

 *where* $a\left(t\right)$ *is IA and* $\phi \left(t\right)$ *is IP.*

Equation (1) is called the amplitude-phase representation of the signal. If the signal $x\left(t\right)$ is MC, it can be written in an analytical amplitude phase form. The following derivative of the phase $\phi \left(t\right)$ with respect to time t is defined as IF:

(2) $$f\left(t\right)=\frac{\phi^{\prime }\left(t\right)}{2\pi }$$

Natural or artificial signals are usually real-valued signals, so it is unrealistic to use Equation (2) to calculate the IF unless the phase function can be effectively extracted from the signal.

To solve this problem, Ville [9] redefined IF as follows, which is also the most popular method to solve the IF of a narrowband signal:

**Definition 2.**  *Let* $z\left(t\right)$ *be the analytic phase-amplitude representation of a given real-valued signal $x\left(t\right)$ in the form of Equation (1), which can be expressed as follows:*

(3) $$z\left(t\right)=a\left(t\right)e^{j\phi \left(t\right)}$$

*where* $a\left(t\right)$ *denotes Analytical Instantaneous Amplitude (AIA), and* $\phi \left(t\right)$ *denotes the Analytical Instantaneous Phase (AIP). Then, the Analytic Instantaneous Frequency (AIF) of the signal is defined as follows:*

(4) $$f\left(t\right)=\frac{1}{2\pi }\frac{d\left[arg\left(z\left(t\right)\right)\right]}{dt}$$

*where* $arg\left(z\left(t\right)\right)$ *represents the phase angle of analytic function* $z\left(t\right)$*. In order to calculate AIF according to Equation (4), the signal* $x\left(t\right)$ *must be analytic.*

According to Boashash’s research [11], for a given signal $x\left(t\right)$, there may be many pairs of $\left[a\left(t\right),\phi \left(t\right)\right]$ or many methods to produce the same signal such as Equation (1). There is no one-to-one correspondence.

However, not all of those pairs $\left[a\left(t\right),\phi \left(t\right)\right]$ are qualified to construct analytic signals like Equation (3). They must satisfy the analytic condition: the signal which can be synthesized by a pair $\left[a\left(t\right),\phi \left(t\right)\right]$ must be narrowband, or the frequency spectrum of amplitude $a\left(t\right)$ must be separated from that of $e^{j\phi \left(t\right)}$ [9]. For MC signals, if the analytical conditions are satisfied, the IF calculated according to Equation (4) is effective and meaningful. The following section discusses the concept of a MC.

### 2.2. Definition of MC and Data-Adaptive Decomposition

The word “mono-component” was originally proposed by Cohen, but he did not give it a specific mathematical formula. Qian [12] gave it a precise mathematical definition.

**Definition 3.**  *A real-valued signal* $x\left(t\right)$ *is MC if with the form in Equation (1) or* *Equation (3), and it should satisfy either $a\left(t\right)\geq 0$* *and $\phi^{\prime }\left(t\right)\geq 0$* *, or $a\left(t\right)\geq 0$* *and $\phi^{\prime }\left(t\right)\leq 0$*.

The definition gives the specific conditions of a MC signal, that is, the phase function $\phi \left(t\right)$ must be monotonically increasing or monotonically decreasing, and the amplitude function $a\left(t\right)$ must be positive. A mono-component signal always has an IF based on Definition 1, but not always an AIF based on Definition 2, unless it satisfies the analytical conditions. Intuitively speaking, a signal is labeled as a MC because it has only one vibration mode, such as a sinusoidal signal or a linear Frequency Modulation signal.

In summary, in order to calculate the IF of component signals, existing methods require that mono-component signals must satisfy Definition 3 and analytical conditions. Therefore, many recent studies have proposed stricter conditions for the adaptive decomposition algorithm, that is, the component signal must be MC and analytic. Based on this analysis and the results of [5,12], the data-adaptive decomposition method is summarized as follows:

**Definition 4.**  *For a given signal $x\left(t\right)$, the data-adaptive composition method decomposes the signal into a sum of several finite components $s_{k}\left(t\right)$* *and a remainder $r\left(t\right)$, that is as follows:*

(5) $$x\left(t\right)=\sum_{k=1}^{K}s_{k}\left(t\right)+r\left(t\right)$$

*where $s_{k}\left(t\right)=a_{k}\left(t\right)cos\left(\phi_{k}\left(t\right)\right)$, with*

(6) $$a_{k}\left(t\right)\geq 0,\;\phi_{k}^{\prime }\left(t\right)\geq 0,\;\forall t$$

*and*

(7) $$H\left[a_{k}\left(t\right)cos\left(\phi_{k}\left(t\right)\right)\right]=a_{k}\left(t\right)H\left[cos\left(\phi_{k}\left(t\right)\right)\right]$$

*where $H\left[\cdot \right]$* *denotes the Hilbert Transform (HT).*

The IF can then be obtained from the analytical form of $a_{k}\left(t\right)e^{j\phi_{k}\left(t\right)}$, as adopted by most researchers. Note that the components $s_{k}\left(t\right)$ satisfying the conditions (6) and (7) are MC signals, but the opposite may be wrong. There is a certain deviation between the HT of a component signal and its quadrature component, which cannot be eliminated even under the narrowband condition. This is not conducive to the calculation of IF. Moreover, the narrowband condition may remove the nonlinear information from the signal, which may be useful and is usually not expected by signal analysis.

Even without considering the problem of HT transformation, the component signals obtained by existing methods do not necessarily satisfy all the conditions in Definition 4. For example, the component signals decomposed by the ADF algorithm are MC, but not necessarily analytical or narrowband. The decomposition results of EMD are not necessarily MC, so occasionally the obtained IFs may be meaningless.

According to Euler’s formula, Equation (3) can be further expanded into the following forms:

(8) $$\begin{array}{ll} z\left(t\right) & =a\left(t\right)e^{j\phi \left(t\right)}\\ & =a\left(t\right)cos\left(\phi \left(t\right)\right)+ja\left(t\right)sin\left(\phi \left(t\right)\right)\\ & =x_{c}\left(t\right)+x_{s}\left(t\right) \end{array}$$

where $x_{c}\left(t\right)=a\left(t\right)cos\left(\phi \left(t\right)\right)$, $x_{s}\left(t\right)=ja\left(t\right)sin\left(\phi \left(t\right)\right)$, and the phase difference between the two signals is 90°, which is called the in-phase component and quadrature component, respectively. According to Equation (8), if the quadrature component can be found directly, HT is not needed, and the component signal does not need to satisfy the analytical conditions, as the AIF can also be calculated directly from Equation (8). However, the following new problems emerge: (1) how to find a pair of $\left[a\left(t\right),\phi \left(t\right)\right]$ to represent signals; and (2) how to find a good method to calculate quadrature components directly without HT.

Fortunately, Huang [5] proposed a new normalization decomposition scheme designated as the NHT, which is an empirical decomposition method to obtain phase-modulated function $cos\left(\phi \left(t\right)\right)$ with unit modulus and corresponding amplitude-modulated function $a\left(t\right)$, respectively. Then, the quadrature component can be calculated directly from the following trigonometric:

(9) $$sin\left(\phi \left(t\right)\right)=\sqrt{1-{cos}^{2}\left(\phi \left(t\right)\right)}$$

Therefore, analyticity is not a necessary condition for calculating the Instantaneous Frequency in NHT. In particular, NHT obtains $\left[a\left(t\right),cos\left(\phi \left(t\right)\right)\right]$ pairs directly, rather than $\left[a\left(t\right),\phi \left(t\right)\right]$ pairs, and the phase function can be computed only after obtaining the quadrature component according to Equation (9). This method has given a reasonable solution to the deficiencies of Definition 4, although without detailed mathematical expressions like EMD. However, it further introduces the normalization iteration process based on EMD sifting, which further increases the computational complexity.

Definition 1 demonstrates that as long as the phase function is known, the IF can be easily calculated from Equation (2). If there is a way to find the phase function and the corresponding amplitude function pairs $\left[a\left(t\right),\phi \left(t\right)\right]$ directly, it will be an ideal alternative. Next, we will introduce a new multi-component decomposition method, which is different from other methods and can directly obtain the phase function.

## 3. Proposed Methods

### 3.1. SFF Definition and Construction

First, a definition of SFF is given as shown below.

**Proposed Definition 1.**  *If there is a pair of real functions* $\left[a\left(t\right),\phi \left(t\right)\right]$*, and it satisfies $a\left(t\right)>0$* *and $\phi^{\prime }\left(t\right)\geq 0$, the signal synthesized by them in the following form is named SFF:*

(10) $$s\left(t\right)=a\left(t\right)sin\left(\phi \left(t\right)\right)$$

Then, the IF of $s\left(t\right)$ can be obtained directly by the derivative of the phase function $\phi \left(t\right)$ as shown in Equation (2), where $a\left(t\right)$ and $\phi \left(t\right)$ are obtained by the proposed SFD algorithm. Notice that SFF is in the form of a quadrature component, and similarly the corresponding in-phase component can be synthesized by the following formula:

(11) $$s\left(t\right)=a\left(t\right)cos\left(\phi \left(t\right)\right)$$

By Definition 3, SFF is inherently MC. Compared with Definition 4, the conditions for calculating IF are much looser, and it is not necessary to consider whether the decomposed component signal is MC or narrowband.

To illustrate how to build SFF, some sequences are presented below.

Let $r\left(t_{k}\right),t_{k}=kT_{s},k=0,1,\cdots ,N-1,T=NT_{s}$ be a given real-valued signal, where N denotes the sample number and $T_{s}$ is the sample time interval. $V_{E}$ denotes the sequence of local extremums as follows:

(12) $$V_{E}≜\left\{{\left(t_{k}^{e},v_{k}^{e}\right)}_{k=1}^{N_{e}}\left|\begin{array}{c} \forall k\in \left[2,N_{e}\right],0<t_{k-1}^{e}<t_{k}^{e}<\left(N-1\right)T_{s}\\ \left(r\left(t_{k}^{e}-T_{s}\right)<r\left(t_{k}^{e}\right),r\left(t_{k}^{e}\right)>r\left(t_{k}^{e}+T_{s}\right)\right)or\\ \left(r\left(t_{k}^{e}-T_{s}\right)>r\left(t_{k}^{e}\right),r\left(t_{k}^{e}\right)<r\left(t_{k}^{e}+T_{s}\right)\right) \end{array}\right.\right\}$$

where $N_{e}$ denotes the number of local extremums, and the pair $t_{k}^{e},v_{k}^{e}$ represents the time and amplitude of the kth local extremum of the signal, respectively.

Let $T_{z}={\left\{t_{k}^{z}\right\}}_{k=1}^{N_{z}}$ represent the zero-crossing time sequence of signal $r\left(t_{k}\right)$, where $N_{z}$ denotes the number of zero-crossing points. Then, a subset $V_{E}^{\prime }$ of the sequence $V_{E}$ is defined, which is represented as follows:

(13) $$V_{E}^{\prime }≜\left\{{\left(t_{k}^{e^{\prime }},v_{k}^{e^{\prime }}\right)}_{k=1}^{N_{e^{\prime }}}\left|t_{k}^{e^{\prime }}\in \left(t_{1}^{Z},t_{k}^{N_{Z}}\right),\right.\left(t_{k}^{e^{\prime }},v_{k}^{e^{\prime }}\right)\in V_{E}\right\}$$

where $N_{e^{\prime }}$ represents the number of local extremum between the first zero-crossing and the last zero-crossing.

Let $P_{e}$ represent the time-phase sequence corresponding to the local extreme points, and $Q_{M}$ denote the time-amplitude sequence of the midpoint of two adjacent local extremum as follows:

(14) $$P_{e}≜\left\{{\left(t_{k}^{e},\phi_{k}^{e}\right)}_{k=1}^{N_{e}}\left|\begin{array}{c} \forall \left(t_{k}^{e},\phi_{k}^{e}\right)\in V_{E},\;\phi_{k}^{e}=\pi \left[\left(k-1\right)+\frac{1}{2}{\left(-1\right)}^{\alpha }\right]\;\\ where\;\alpha =\left\{\begin{array}{c} \begin{array}{c} 0\;{\;\;\;v}_{k-1}^{e}>v_{k}^{e},\;\forall k\in \left[2,N^{e}\right]\\ 1\;{\;\;\;v}_{k-1}^{e}<v_{k}^{e},\;\forall k\in \left[2,N^{e}\right] \end{array}\\ \begin{array}{c} 0{\;\;\;v}_{1}^{e}<r\left(t_{1}^{e}-T_{s}\right)\;\;\;\;\;\;\;\;\;\;\;\;\;\;\;\;\\ 1{\;\;\;v}_{1}^{e}>r\left(t_{1}^{e}-T_{s}\right)\;\;\;\;\;\;\;\;\;\;\;\;\;\;\;\; \end{array} \end{array}\right. \end{array}\right.\right\}$$

(15) $$Q_{M}≜\left\{{\left(t_{k}^{m},a_{k}^{m}\right)}_{k=1}^{N_{e}-1}\left|\begin{array}{c} \forall \left(t_{k}^{e},\phi_{k}^{e}\right)\in V_{E},\\ t_{k}^{m}=\frac{t_{k}^{e}+t_{k+1}^{e}}{2},a_{k}^{m}=\frac{\left|v_{k}^{e}-v_{k+1}^{e}\right|}{2} \end{array}\right.\right\}$$

where α is the sign factor, the pair $t_{k}^{e},\phi_{k}^{e}$ is the time index, and the phase corresponding to the local extreme in Equation (12), respectively.

Taking the phase sequence $P_{e}$ and amplitude sequence $Q_{M}$ defined above in Equations (14) and (15) as anchor nodes, the Hermite interpolation method [33] with shape preserving is used for interpolation. This method can keep the non-negativity and monotonicity of the fitting function by adjusting the first-order derivative of nodes, or mapping it to the Fritsch-Carlson monotonicity region or Butland region [34,35]. Using cubic Hermite polynomial interpolation, we can obtain a pair of fitted IP and IA functions, which are expressed by $\phi \left(t\right)$ and $a\left(t\right)$, respectively.

Next, we will prove that the fitted IP $\phi \left(t\right)$ and IA $a\left(t\right)$ function can satisfy the conditions of Proposed Definition 1.

Let the time sequence ${\left\{t_{k},t_{k}=t_{k}^{e}\right\}}_{k=1}^{N_{e}}$ be the *x*-axis coordinate node, and ${\left\{\phi_{k},\phi_{k}=\phi_{k}^{e}\right\}}_{k=1}^{N_{e}}$ be the corresponding phase value on the *y*-axis. The local time interval is expressed as $∆t_{k+0.5}=t_{k+1}^{e}-t_{k}^{e}$, and the phase difference in adjacent nodes is expressed as $∆\phi_{k+0.5}=\phi_{k+1}^{e}-\phi_{k}^{e}$, then the slope of adjacent nodes can be denoted by $∆p_{k+0.5}=∆\phi_{k+0.5}/∆t_{k+0.5}$.

According to Equation (14), $0<t_{k-1}^{e}<t_{k}^{e}<\left(N-1\right)T_{s}$ and $\phi_{k-1}^{e}<\phi_{k}^{e}$, then the product $∆p_{k+0.5}∆p_{k-0.5}>0$, so the $\phi_{k}$ is monotonically increasing on set ${\left\{t_{k}\right\}}_{k=1}^{N_{e}}$.

In order to make the interpolation curve also increase monotonically, the first derivative of each node is estimated according to the finite difference method in reference [27], expressed as ${\dot{p}}_{k}$; if ${\dot{p}}_{k}$ does not fall into the so-called monotone region (Butland region), the value of ${\dot{p}}_{k}$ needs to adjust according to the following equation:

(16) $${\dot{p}}_{k}=\frac{∆p_{k+0.5}∆p_{k-0.5}}{∆p_{k+0.5}+∆p_{k-0.5}}$$

Otherwise, if it falls into the monotone region, the ${\dot{p}}_{k}$ will remain unchanged. This strategy can guarantee that the fitted curve $\phi \left(t\right)$ is monotonically increasing after the cubic Hermite polynomial interpolation, and its first derivative is first-order continuous. So, $\phi^{\prime }\left(t\right)>0,\forall t\in \left[0,T\right]$.

Similarly, let the time sequence ${\left\{t_{k},t_{k}=t_{k}^{m}\right\}}_{k=1}^{N_{e}-1}$ be the x-axis coordinate node, and let ${\left\{a_{k},a_{k}=a_{k}^{m}\right\}}_{k=1}^{N_{e}-1}$ be the corresponding amplitude value on the y-axis. The local time interval is expressed as $∆t_{k+0.5}=t_{k+1}^{m}-t_{k}^{m}$, and the amplitude difference in adjacent nodes is expressed as $∆a_{k+0.5}=a_{k+1}^{m}-a_{k}^{m}$, then the adjacent slope is denoted by $∆q_{k+0.5}=∆a_{k+0.5}/∆t_{k+0.5}$. Let ${\dot{q}}_{k}$ be the first derivative of the kth node, which can be calculated by the finite difference method. The constraint method [6] ensures the non-negativity of the interpolation result of cubic Hermite polynomials, mainly as follows.

If $\left(-3a_{k}/∆t_{k+0.5}\right)\leq {\dot{q}}_{k}\leq \left(3a_{k}/∆t_{k-0.5}\right)$, leave ${\dot{q}}_{k}$ unchanged, otherwise let

(17) $${\dot{q}}_{k}\leftarrow min\left(\frac{3a_{k}}{∆t_{k+0.5}},max\left(\frac{-3a_{k}}{∆t_{k-0.5}},{\dot{q}}_{k}\right)\right)$$

In this way, after the piecewise cubic Hermite polynomial interpolation, the fitted curve $a\left(t\right)$ has the same sign as the piecewise linear interpolation result. According to Equation (15), $a_{k}>0,\;\forall k\in \left[1,N_{e}-1\right]$, so the piecewise linear interpolation result is non-negative, which means that the piecewise cubic polynomial interpolation result is also non-negative, that is, $a\left(t\right)>0$.

The fitted amplitude $a\left(t\right)$ and phase $\phi \left(t\right)$ by the above methods are synthesized according to Equation (10), then the SFF $s\left(t\right)$ can be constructed as follows:

(18) $$s\left(t\right)=a\left(t\right)sin\left(\phi \left(t\right)\right)$$

Obviously, both of the synthesized functions $s\left(t\right)$ satisfy the conditions for the amplitude and phase functions in Proposed Definition 1, so they are SFFs. SFD uses an iterative method to decompose initial signals into SFF components, such as Equation (18). The definition and algorithm of the SFD will be given in the next section.

### 3.2. SFD Definition and Algorithm

In the above section, the construction method of SFF is given. The next key is the process of decomposing SFF components from multi-component signals. Next, we will introduce the definition and algorithm of SFD.

**Proposed Definition 2.**  *For a given real signal $x\left(t\right)$, the SFD method decomposes it into a sum of several SFF components $s_{k}\left(t\right)$, trend components $m_{j}\left(t\right)$, and a remainder signal $r\left(t\right)$, that is as follows:*

(19) $$x\left(t\right)=\sum_{k=1}^{N_{s}}s_{k}\left(t\right)+\sum_{j=1}^{N_{m}}m_{j}\left(t\right)+r\left(t\right)$$

*where $N_{s}$* *and $N_{m}$* *represent the number of SFF components and trend components, respectively.*

The trend components usually are the direct current component or the low-frequency components in the given signal. They have very low oscillation frequencies relative to the SFF component, which represents the total deviation of the signal from the abscissa.

By Proposed Definition 2, SFD is an iterative algorithm. For each iteration, an SFF function is constructed from the input signal and then subtracted from the input signal. The same procedure is repeated on the residual function, just like EMD, until the decomposition times are greater than the preset decomposition order or the residual energy is less than the preset threshold. The details of SFD are shown in the following chart:

The parameters ε and ξ in Algorithm 1 can be set according to the actual signal.
**Algorithm 1** Proposed SFD algorithm**Input:**$\mathrm{The}\;\mathrm{decomposable}\;\mathrm{signal}\;x\left(t\right)$, the order of SFD λ, and the residual energy threshold ε and ξ.**Output**:$\mathrm{SFFs}\;s_{k}\left(t\right)$$,\;\mathrm{Ias}\;a_{k}\left(t\right)$$,\;\mathrm{IPs}\;\phi_{k}\left(t\right)$$,\;\mathrm{IFs}\;f_{k}\left(t\right)$$,\;\mathrm{the}\;\mathrm{mean}\;\mathrm{components}\;m_{j}\left(t\right)$$\;\mathrm{and}\;\mathrm{the}\;\mathrm{residual}\;\mathrm{component}\;r\left(t\right)$.1 $\mathrm{Initialize}:\;r\left(t\right)=x\left(t\right),\;k=0,\;j=0,\;p_{x}=p_{r}=10log\left(\sum x^{2}\left(t\right)/N\right)$. Set the decomposition time k=0, the initial values for constants λ, ε and ξ$,\;\mathrm{and}\;\mathrm{the}\;\mathrm{residual}\;\mathrm{component}\;r^{1}\left(t\right)=0$.2 $\mathrm{Compute}\;1:\;\mathrm{Count}\;\mathrm{the}\;\mathrm{number}\;N_{e}$$\;\mathrm{of}\;\mathrm{local}\;\mathrm{extremum}\;\mathrm{of}\;r\left(t\right)$$\;\mathrm{according}\;\mathrm{to}\;\mathrm{Equation}\;(12),\;\mathrm{the}\;\mathrm{number}\;N_{e^{\prime }}$$\;\mathrm{according}\;\mathrm{to}\;\mathrm{Equation}\;(13),\;\mathrm{the}\;\mathrm{number}\;N_{z}$$\;\mathrm{of}\;\mathrm{zero}-\mathrm{crossing}\;\mathrm{points},\;\mathrm{and}\;\mathrm{let}\;T_{1}=N_{e},\;T_{2}=N_{e^{\prime }},\;T_{3}=N_{z}$.3 $\mathrm{while}\;0\;T_{1}\geq 5$$\;\mathrm{and}\;p_{x}-p_{r}<\varepsilon$ and k<λ **do**4 
**Operation I: Construct mean function**5 
$\mathrm{Determine}\;\mathrm{all}\;\mathrm{local}\;\mathrm{maxima}\;\mathrm{and}\;\mathrm{minima}\;\mathrm{of}\;\mathrm{signal}\;r\left(t\right)$, and fit the upper and lower envelopes by the cubic spline interpolation method, respectively.6 
$\mathrm{Calculate}\;\mathrm{the}\;\mathrm{mean}\;\mathrm{component}\;m^{1}\left(t\right)$ of the upper envelope and lower envelope.7 
$r_{1}\left(t\right)=r\left(t\right)-m^{1}\left(t\right)$8 
$\mathrm{Operation}\;\mathrm{II}:\;\mathrm{Construct}\;\mathrm{SFF}\;\mathrm{from}\;r_{1}\left(t\right)$9 
$\mathrm{Carry}\;\mathrm{out}\;\mathrm{Compute}\;I\;\mathrm{on}\;\mathrm{the}\;r_{1}\left(t\right)$$,\;\mathrm{and}\;\mathrm{obtain}\;T_{1},\;T_{2},\;T_{3}$10 
$\mathrm{while}\;1\;T_{2}\neq T_{3}-1$$\;\mathrm{or}\;\left|T_{1}-T_{3}\right|>1$ **do**11 

$\mathrm{Carry}\;\mathrm{out}\;\mathrm{Operation}\;I\;\mathrm{on}\;\mathrm{the}\;r_{1}\left(t\right)$$,\;\mathrm{and}\;\mathrm{obtain}\;\mathrm{the}\;\mathrm{mean}\;\mathrm{component}\;m^{2}\left(t\right)$$\;\mathrm{and}\;r_{2}\left(t\right)$$,\;\mathrm{where}\;r_{2}\left(t\right)=r_{1}\left(t\right)-m^{2}\left(t\right)$12 

$r_{1}\left(t\right)=r_{2}\left(t\right)$13 

$\mathrm{Carry}\;\mathrm{out}\;\mathrm{Compute}\;1\;\mathrm{on}\;\mathrm{the}\;r_{1}\left(t\right)$$,\;\mathrm{and}\;\mathrm{obtain}\;T_{1},\;T_{2},\;T_{3}$14 
**end while 1**15 
$\mathrm{Construct}\;V_{E}$$\;\mathrm{on}\;\mathrm{the}\;\mathrm{residual}\;r_{1}\left(t\right)$ according to Equation (12).16 
$\mathrm{Construct}\;P_{e}$$\;\mathrm{according}\;\mathrm{to}\;\mathrm{Equation}\;(14)\;\mathrm{and}\;Q_{M}$ according to Equation (15).17 
k=k+118 
$\mathrm{Interpolation}\;\mathrm{on}\;P_{e}$$\;\mathrm{and}\;Q_{M}$$,\;\mathrm{obtain}\;\mathrm{fitted}\;\mathrm{IP}\;\phi_{k}\left(t\right)$$\;\mathrm{and}\;\mathrm{IA}\;a_{k}\left(t\right)$19 
$\mathrm{Compute}\;\mathrm{IF}\;\mathrm{by}\;f_{k}\left(t\right)=d\phi_{k}\left(t\right)/dt$20 
$\mathrm{Construct}\;\mathrm{SFF}\;\mathrm{by}\;a_{k}\left(t\right)$$\;\mathrm{and}\;\phi_{k}\left(t\right)$$\;\mathrm{according}\;\mathrm{to}\;\mathrm{Equation}\;(18),\;\mathrm{and}\;\mathrm{let}\;s_{k}\left(t\right)=a_{k}\left(t\right)sin\left(\phi_{k}\left(t\right)\right)$21 
$r_{2}\left(t\right)=r_{1}\left(t\right)-s_{k}\left(t\right)$22 
$r^{1}\left(t\right)=r^{1}\left(t\right)+r_{2}\left(t\right)$23 
$\mathrm{Operation}\;\mathrm{III}:\;\mathrm{Construct}\;\mathrm{SFF}\;\mathrm{from}\;m^{1}\left(t\right)$24 
$\mathrm{Count}\;\mathrm{local}\;\mathrm{extremum}\;\mathrm{number}\;N_{e}$$\;\mathrm{of}\;m^{1}\left(t\right)$$\;\mathrm{and}\;\mathrm{let}\;T_{1}=N_{e}$25 
$\mathrm{while}\;2\;T_{1}>3$ and k<λ **do**26 

$\mathrm{Carry}\;\mathrm{out}\;\mathrm{Operation}\;I\;\mathrm{on}\;\mathrm{the}\;\mathrm{mean}\;\mathrm{component}\;m^{1}\left(t\right)$$\;\mathrm{then}\;\mathrm{obtain}\;r_{2}\left(t\right)$$\;\mathrm{and}\;m^{2}\left(t\right)$27 

$p_{r}=10log\left(\sum r_{2}^{2}\left(t\right)/N\right)$28 

$\mathrm{if}\;p_{r}>p_{x}-\xi$ and k<λ29 


$\mathrm{Carry}\;\mathrm{out}\;\mathrm{Operation}\;\mathrm{II}\;\mathrm{on}\;\mathrm{the}\;\mathrm{signal}\;r_{2}\left(t\right)$$,\;\mathrm{and}\;\mathrm{then}\;\mathrm{obtain}\;\mathrm{IA}\;a_{k}\left(t\right)$$,\;\mathrm{IP}\;\phi_{k}\left(t\right)$$,\;\mathrm{IF}\;f_{k}\left(t\right)$$,\;\mathrm{SFF}\;s_{k}\left(t\right)$$,\;\mathrm{and}\;\mathrm{residual}\;\mathrm{signal}\;r_{3}\left(t\right)$30 


$r_{2}\left(t\right)=r_{3}\left(t\right)$31 

**else**32 


k=k+133 

**end if**34 

$r^{1}\left(t\right)=r^{1}\left(t\right)+r_{2}\left(t\right)$35 

$m^{1}\left(t\right)=m^{2}\left(t\right)$36 

$\mathrm{Count}\;\mathrm{local}\;\mathrm{extreme}\;\mathrm{number}\;N_{e}$$\;\mathrm{of}\;m^{1}\left(t\right)$$,\;T_{1}=N_{e}$37 
**end while 2**38 
j=j+139 
$m_{j}\left(t\right)=m^{1}\left(t\right),\;r\left(t\right)=r^{1}\left(t\right)$40 
$\mathrm{Carry}\;\mathrm{out}\;\mathrm{Compute}\;1\;\mathrm{on}\;\mathrm{the}\;\mathrm{residual}\;r\left(t\right)$$,\;\mathrm{obtain}\;T_{1},\;T_{2},\;T_{3}$41 
$p_{r}=10log\left(\sum r_{2}^{2}\left(t\right)/N\right)$42 **end while 0**

### 3.3. Sinusoidal Fitting Spectrum

According to the proposed definition of SFFs in Equation (10) and Algorithm 1, IF and IA are easily obtained from the decomposed SFFs. The several decomposed SFFs can also be expressed in the time-frequency plane and is called Sinusoidal Fitting Spectrum (SFS), as shown in the following equation:

(20) $$A\left(t,f\right)=SFT\left[x\left(t\right)\right]=\left\{\begin{array}{cc} 0 & \forall k\in \left[1,N_{s}\right],\;f\left(t\right)\neq f_{k}\left(t\right)\\ \sum_{k}a_{k}\left(t\right) & for\;all\;k\;when\;f\left(t\right)=f_{k}\left(t\right) \end{array}\right.$$

where $t\in \left[0,T\right]$ denotes the time, and $f_{k}\left(t\right)=d\phi_{k}\left(t\right)/dt,k=1,\cdots ,N_{s}$ is the kth IF.

Inversely, the original signal can be reconstructed from the instantaneous time-frequency-amplitude spectra, that is as follows:

(21) $$\hat{x}\left(t\right)={SFT}^{-1}\left[A\left(t,f\right)\right]≜\sum_{k=1}^{N_{s}}a_{k}\left(t\right)sin\left(2\pi f_{k}t\right)$$

where the operators $SFT\left[\cdot \right]$ and ${SFT}^{-1}\left[\cdot \right]$ denote a Sine Fitting Transform (SFT) and its inverse transform, respectively. $N_{s}$ is the order number of SFD, and the signal $\hat{x}\left(t\right)$ is the approximation of the initial signal $x\left(t\right)$.

## 4. Experiment Results and Discussion

In this section, we will use some simulation data and actual data processing to verify the effectiveness of our method. The intermediate process of algorithm processing will be given, from which readers can observe the details of the algorithm processing, and finally the SFSs of these signals are shown.

### 4.1. Multi-Componential Signal Separation

**Experiment 1:**  *The signal* $x_{sig1}\left(t\right)$ *is a combined signal, which is composed of three signal components, and its mathematical expression is as follows:*

(22) $$x_{sig1}\left(t\right)=6t^{2}+cos\left(4\pi t+10\pi t^{2}\right)+\left\{\begin{array}{c} cos\left(100\pi t-10\pi \right),t>0.5\\ cos\left(60\pi t\right),t\leq 0.5 \end{array}\right.$$

*where* $t\in \left[0,1\right]$*. In* $x_{sig1}\left(t\right)$*, there are three different items with different vibration modes: the first item is a second-order trend component with a bigger amplitude, the second one is a linear modulated frequency component with unit amplitude, and the third component is a segmented signal composed of two CW signals of different frequencies.*

**Figure 1:**
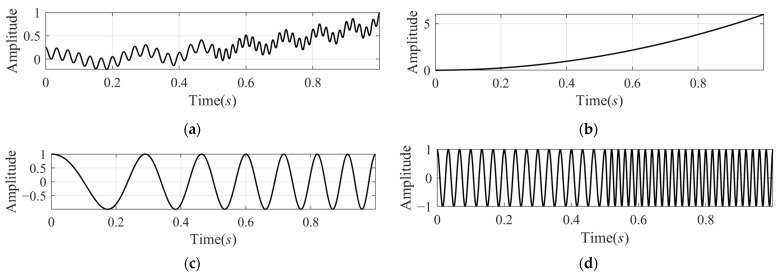
Mono-component signal $x_{sig1}\left(t\right)$ and its components: (**a**) the signal $x_{sig1}\left(t\right)$; (**b**) the second order trend component $6t^{2}$; (**c**) the linear modulated frequency component $cos\left(4\pi t+10\pi t^{2}\right)$; and (**d**) the segmented component composed of two CW signals $cos\left(100\pi t-10\pi \right),t>0.5$ and $cos\left(60\pi t\right),\;t\leq 0.5$.

**Figure 2:**
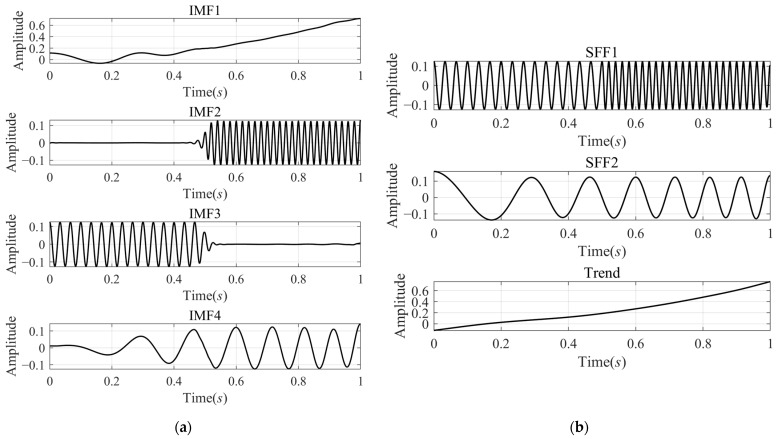
Comparison of decomposition results of signal $x_{sig1}\left(t\right)$ by VMD and SFD: (**a**) four IMFs decomposed by VMD; and (**b**) two SFFs and trend component decomposed by SFD.

Figure 1 shows the signal $x_{sig1}\left(t\right)$ and its three components separately, and Figure 2 shows the decomposition results of VMD and SFD. In Figure 2, the left column shows the IMFs decomposed by the VMD. IMF1 and IMF4 correspond to the first and second components. The third component, two segmented signals with different frequencies, was decomposed into two IMFs, IMF2 and IMF3, due to the fact that the VMD will adaptively decompose the original signal into some IMFs of different frequency bands without mode mixing. The right column shows the decomposition results of SFD, including two SFFs and the trend component. SFF1 and SFF2 correspond to Figure 1c,d. The trend component is similar to Figure 1b, but there is some obvious error in the first 0.1 s.

**Figure 3:**
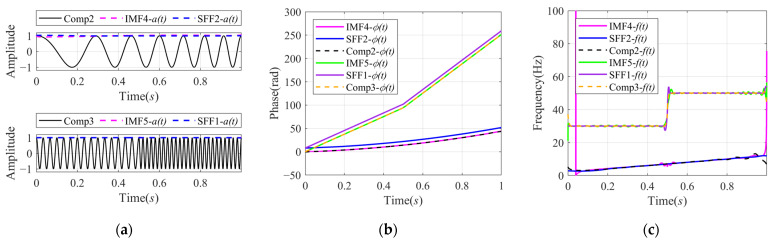
Comparison of instantaneous characteristics of signal $x_{sig1}\left(t\right)$ calculated by VMD and SFD: (**a**) Second and third original components (solid line) of $x_{sig1}\left(t\right)$ and their corresponding AIAs (pink dashed line) calculated by VMD and IAs (blue dashed line) calculated by SFD; (**b**) AIPs of second and third components of $x_{sig1}\left(t\right)$, AIPs of IMFs calculated by VMD, and IPs of SFFs calculated by SFD; and (**c**) AIFs of second and third components of $x_{sig1}\left(t\right)$, AIFs of IMFs calculated by VMD, and IFs of SFFs calculated by SFD.

We have carried out the instantaneous characteristics calculated from the decomposition results by SFD and VMD, as shown in Figure 3. For better comparison, we added IMF2 and IMF3 to make a new component labeled as IMF5, and the instantaneous characteristics of the IMFs were calculated using Hilbert transformation and an analytical function. The instantaneous characteristics of the second and third components of $x_{sig1}\left(t\right)$, shown as Comp2 and Comp3 in Figure 3, were also calculated using Hilbert transformation. The instantaneous characteristics of the SFFs were directly calculated from the SFD method.

In order to facilitate the comparison and analysis, the extracted Instantaneous Amplitude together with the corresponding actual components are shown in Figure 3a. In the top panel of Figure 3a, we can see that the $a\left(t\right)$ of SFF2 is flatter and closer to the actual envelope than the $a\left(t\right)$ of IMF4. According to Figure 2a, it may be because some low-frequency components of the IMF4 were decomposed into other IMFs, such as IMF1.

Figure 3b shows a comparison of the IPs obtained by SFD, the AIPs calculated from the IMFs of VMD, and the AIPs of the actual components. The AIPs of IMFs extracted by VMD are closer to the AIPs of the actual components, while there is a relatively stable error between the IPs of the SFFs and the actual AIPs due to the different initial phases. Therefore, the proposed method can accurately estimate the trend of phase change instead of the accurate phase value. This conclusion can also be verified by the results of Instantaneous Frequency extraction.

Figure 3c shows a comparison between the IFs of SFFs, the AIFs of IMFs, and the AIFs of the actual components. The IFs obtained by the two decomposition methods are consistent with the actual AIFs, but there are also some fluctuation errors, especially at the frequency mutation and the beginning and the end of the signal. Compared with the results of VMD, the IFs of SFF are relatively smooth and accurate at the beginning and the end of the signal.

**Experiment 2.**  *The second tested signal* $x_{sig2}\left(t\right)$ *[5]* *is a damped duffing model with a chirp frequency, and its expression has the following form:*

(23) $$x_{sig2}\left(t\right)=exp\left(-\frac{t}{256}\right)cos\left[\frac{\pi }{64}\left(\frac{t^{2}}{512}+32\right)+0.3sin\left(\frac{\pi }{32}\left(\frac{t^{2}}{512}+32\right)\right)\right]$$

**Figure 4:**
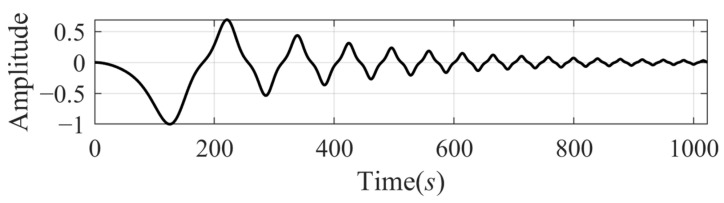
The damped duffing signal $x_{sig2}\left(t\right)$.

*with* $t\in \left[0,1024\right]$*. The signal is shown in Figure 4*.

Before the decomposition experiment, we make the following brief analysis: let $a\left(t\right)=exp\left(-t/256\right)$, $\phi_{1}\left(t\right)=\pi \left(t^{2}/512+32\right)/64$, $\phi_{2}\left(t\right)=\pi \left(t^{2}/512+32\right)/32$, $\phi_{0}\left(t\right)=\phi_{1}\left(t\right)+0.3sin\left(\phi_{2}\left(t\right)\right)$.Then Equation (23) can be written as follows:

(24) $$x_{sig2}\left(t\right)=a\left(t\right)cos\left(\phi_{0}\left(t\right)\right)=a\left(t\right)cos\left(\phi_{1}\left(t\right)+0.3sin\left(\phi_{2}\left(t\right)\right)\right)$$

We can regard the second term and the third term in Equation (24) as the AM-FM expression and the AM-FM-PM expression of the signal, respectively. So we may guess that the result of the multi-component decomposition may be only one component in the case corresponding to the second term. The IF is the derivative of $\phi_{0}\left(t\right)$, that is $\phi_{0}^{\prime }\left(t\right)$, and the IF is a sine wave superimposed on a linear frequency. Or there may be two components, corresponding to $\phi_{1}\left(t\right)$ and $\phi_{2}\left(t\right)$, respectively, and the corresponding IFs can also be obtained by their derivatives.

**Figure 5:**
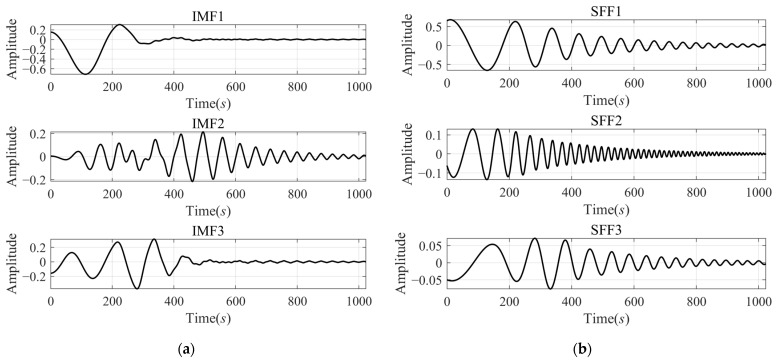
Comparison of decomposition results of signal $x_{sig2}\left(t\right)$ by VMD and SFD: (**a**) IMFs decomposed by VMD; and (**b**) SFFs decomposed by SFD.

The decomposition results of $x_{sig2}\left(t\right)$ by VMD and SFD are shown in Figure 5. As is shown, the two methods both decomposed the signal into three components. All the IMFs calculated by VMD have large errors at the beginning time and after 500s, which means they lost both the low-frequency component at the beginning and the high-frequency components of the second half. The decomposition results by SFD have a better performance. Compared with the results decomposed by VMD, the SFD method misses a small amount of low-frequency components and retains more high-frequency components.

**Figure 6:**
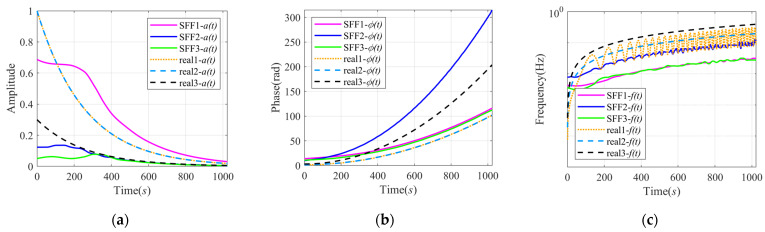

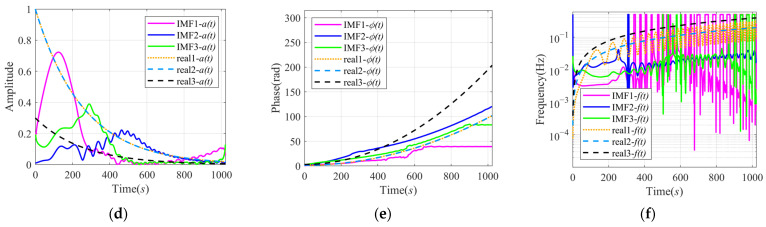
Comparison of instantaneous characteristics of signal $x_{sig2}\left(t\right)$ calculated by VMD and SFD: (**a**) comparison of IAs of SFFs and AIAs of actual components; (**b**) comparison of IPs of SFFs and AIPs of actual components; (**c**) comparison of IFs of SFFs and AIFs of actual components; (**d**) comparison of AIAs of IMFs and actual components; (**e**) comparison of AIPs of IMFs and actual components; and (**f**) comparison of AIFs of IMFs and actual components.

Figure 6 shows the comparison of the instantaneous characteristics obtained by SFD and VMD, compared with the instantaneous characteristics of actual components. The real1- represents the characteristics of the AM-FM signal, and hence real1-$a\left(t\right)$, real1-$\phi \left(t\right)$, and real1-$f\left(t\right)$, respectively, represent $a\left(t\right)$, $\phi_{0}\left(t\right)$, and $\phi_{0}^{\prime }\left(t\right)$. The real2- and real3- represent the characteristics of two components of the AM-FM-PM signal. The real2-$a\left(t\right)$ and real3-$a\left(t\right)$, respectively, represent $a\left(t\right)$ and 0.3*a*(*t*). Moreover, the real2-$\phi \left(t\right)$ and real3-$\phi \left(t\right)$, respectively, represent $\phi_{1}\left(t\right)$ and $\phi_{2}\left(t\right)$, and the real2-$f\left(t\right)$ and real3-$f\left(t\right)$ are the derivatives of the above phase. Therefore, the curve real1-$a\left(t\right)$ and real2-$a\left(t\right)$ overlap in Figure 6a,d, and the curve real1-$\phi \left(t\right)$ and real2-$\phi \left(t\right)$ approximately coincide in Figure 6b,e.

In Figure 6a–c, the curves of SFF2- and SFF3- approximately coincide, so they may be decomposed from a certain component. In Figure 6a, the curve SFF1-$a\left(t\right)$ is relatively closer to real1-$a\left(t\right)$ and real2-$a\left(t\right)$, and the SFF2-$a\left(t\right)$ and SFF3-$a\left(t\right)$ are closer to real3-$a\left(t\right)$. The amplitudes of SFF2 and SFF3 are much different from the real3-$a\left(t\right)$ at the beginning, which may be caused by the slow change in the amplitude at the starting time shown in Figure 5. The curves SFF1-$\phi \left(t\right)$, SFF3-$\phi \left(t\right)$, and real2-$\phi \left(t\right)$ are parallel, and the curve SFF2-$\phi \left(t\right)$ and real3-$\phi \left(t\right)$ are approximately parallel, with an interval that increases with time. Therefore, the signal $x_{sig2}\left(t\right)$ may be regarded as an AM-FM-PM signal when using SFD. The first and third SFF may be divided from the component whose amplitude is $a\left(t\right)$ and phase is $\phi_{1}\left(t\right)$, and the second SFF corresponds to the component whose amplitude is $0.3a\left(t\right)$ and phase is $\phi_{2}\left(t\right)$. This speculation is confirmed in Figure 6c. The curves SFF1-$f\left(t\right)$ and SFF3-$f\left(t\right)$ are parallel to the curve real2-$f\left(t\right)$, and the curve SFF2-$f\left(t\right)$ is parallel to the higher curve real3-$f\left(t\right)$. Although there has always been a certain error between them, the values of the errors are all relatively small and the maximum is around 0.3 Hz.

The signal was decomposed by VMD into three IMFs. Although the amplitudes of the three IMFs are, respectively, approximate to real2-$a\left(t\right)$ and real3-$a\left(t\right)$ after 500 s in Figure 6d, overall there is a large error. It is also hard to find a correspondence between the VMD results and the real components in terms of phase and frequency in Figure 6e,f. The obvious oscillation of IFs after 300 s in Figure 6f is caused by the non-monotonicity of the IPs. Therefore, the decomposition results of VMD are unreasonable, and this method failed in this experiment.

### 4.2. SFD for Real Signal

**Figure 7:**
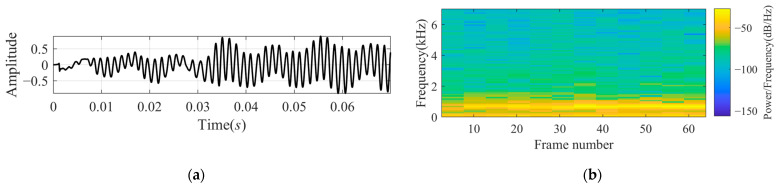
A portion of the real signal recorded from a sonar target echo after bandpass filtering: (**a**) waveform $x_{sigR}\left(t\right)$; and (**b**) time-frequency spectrum.

**Experiment 3:**  *We recorded a sonar target echo signal and intercepted a piece to proceed named* $x_{sigR}\left(t\right)$. *Before decomposing, the signal passes through a band-pass FIR filter of 100–7000 kHz. The waveform and its time-frequency spectrum are shown in Figure 7. The echo is a narrowband signal with around 700 Hz. In Figure 7, we can easily observe that there is no echo at the beginning of around 0.01 s.*

**Figure 8:**
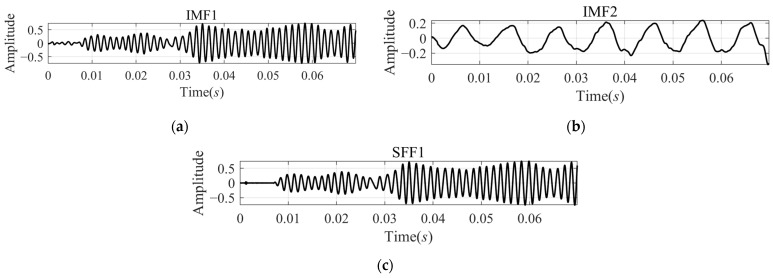
Comparison of decomposition results of signal $x_{sigR}\left(t\right)$ by VMD and SFD: (**a**) IMF1 calculated by VMD; (**b**) IMF2 calculated by VMD; and (**c**) SFF calculated by SFD.

The decomposition results from VMD are shown in Figure 8a,b, and the results from SFD are shown in Figure 8c. The IMF1 and the SFF1 look like the echo signal and the amplitude fluctuation seem to be caused by multipath effects [17].

**Figure 9:**
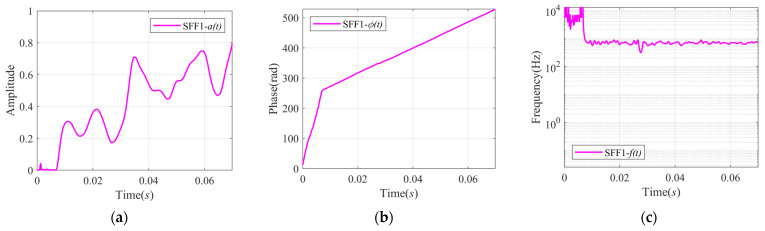

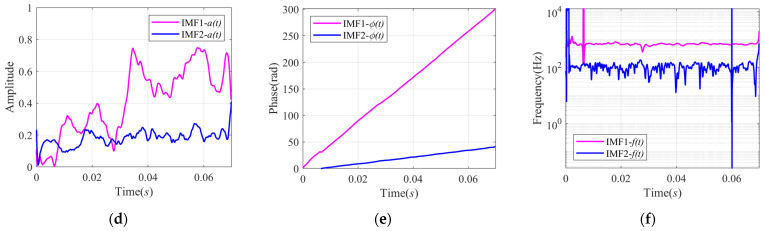
Comparison of instantaneous characteristics of signal $x_{sigR}\left(t\right)$ calculated by VMD and SFD: (**a**) IA of SFF; (**b**) IP of SFF; (**c**) IF of SFF; (**d**) AIAs of IMFs; (**e**) AIPs of IMFs; and (**f**) AIFs of IMFs.

The comparison of the instantaneous characteristics estimated by SFD and VMD is shown in Figure 9. In Figure 9a,d, the Instantaneous Amplitude of SFF1 and IMF1 are similar, and they both have some valleys at the same intervals. The changes in the amplitude are consistent with the change in the echo power in the time-frequency spectrum.

In Figure 9c,f, the frequencies of SFF1 and IMF1 are around 700 Hz, which further proves that these two components are echo components. Some peaks appear at the beginning time corresponding to the first valley of the amplitude in Figure 9c, which is because there is no echo here and hence there is a phase mutation at around 0.0075 s in Figure 9b. However, this phenomenon is not reflected in Figure 9f. The VMD method does not separate the echo component from some similar noise completely compared with SFD, but a strong low-frequency noise is also decomposed by VMD. In general, the proposed method can accurately obtain the mono-component from the original signal, and the calculated instantaneous parameters are more accurate and smoother.

### 4.3. SFSs of the Above Signals

**Figure 10:**
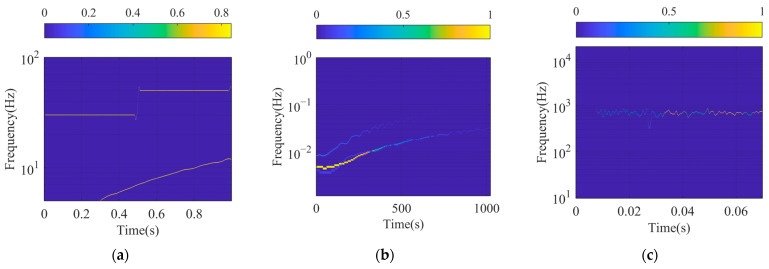
SFSs of different signals: (**a**) SFS of $x_{sig1}\left(t\right)$; (**b**) SFS of $x_{sig2}\left(t\right)$; and (**c**) SFS of $x_{sigR}\left(t\right)$.

**Figure 11:**
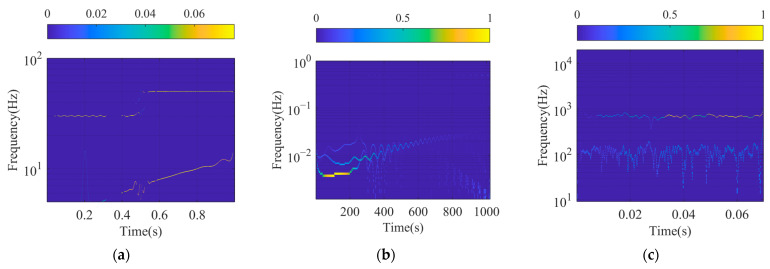
HS of different signals: (**a**) HS of $x_{sig1}\left(t\right)$; (**b**) HS of $x_{sig2}\left(t\right)$; and (**c**) HS of $x_{sigR}\left(t\right)$.

The SFSs of signal $x_{sig1}\left(t\right)$, $x_{sig2}\left(t\right)$, and $x_{sigR}\left(t\right)$ are shown in Figure 10 and are compared with the Hilbert spectrum (HS) of VMD as shown in Figure 11. It is easy to observe the IF of the signal component and the corresponding IA from the spectrum. The spectrum curves are quite slim, which shows good TF resolution and can also identify the strength of the frequency at any point of the SFS.

The processing results of $x_{sig1}\left(t\right)$, shown in Figure 10a and Figure 11a, are similar. Both of them demonstrate that there are two CW components of different frequencies and an FM signal. The instantaneous components of $x_{sig1}\left(t\right)$ extracted by SFD are smoother, and the amplitude is relatively stationary compared with the results contracted by VMD. There is more fluctuation at 0.5s in Figure 11a because of the bad performance of Hilbert transformation when processing the mutation or the ends of the signal.

The results of $x_{sig2}\left(t\right)$ and $x_{sigR}\left(t\right)$ extracted by SFD are better than those of VMD. Although the obtained frequency components are similar, the spectra calculated by SFM are clearer and the corresponding Instantaneous Frequency curves are smoother.

## 5. Conclusions

In this paper, a new MC signal model called SFF, and a new method of multi-component signal decomposition called SFD is proposed. The IAs and IPs are fitted by using the extreme values of the signal and the corresponding time indices, the SFF components are further synthesized by them, and the IFs can also be calculated directly according to the derivative of the IPs. Through iterative processing, the signal can be decomposed into multiple SFF components, trend components, and residual components. The proposed method does not need a sifting operation, which is conducive to real-time operation and processing, and the acquisition of instantaneous parameters is simpler and more direct. According to the simulations and actual signal processing results, SFD can obtain more reasonable results, and the fitting curves of instantaneous parameters are smoother. It can obtain a high resolution in the time-frequency domain at the same time, so the real-time changes in frequency and corresponding amplitude can be observed on SFS, which is of great significance for further analysis and understanding of the signal composition and information.

## Data Availability

The data are available from the corresponding author upon reasonable request.

## References

[1] Hlawatsch F., Matz G., Hlawatsch F., Auger F. (2008). Time-Frequency Methods for Non-stationary Statistical Signal Processing. Time-Frequency Analysis: Concepts and Methods.

[2] Wu Y., Li X. (2017). Elimination of cross-terms in the Wigner–Ville distribution of multi-component LFM signals. IET Signal Process..

[3] Allen J.B., Rabiner L.R. (1977). A unified approach to short-time Fourier analysis and synthesis. Proc. IEEE.

[4] Portnoff M. (1980). Time-frequency representation of digital signals and systems based on short-time Fourier analysis. IEEE Trans. Acoust. Speech Signal Process..

[5] Huang N.E., Wu Z., Long S.R., Arnold K.C., Chen X., Blank K. (2009). On instantaneous frequency. Adv. Adapt. Data Anal..

[6] Carson J.R., Fry T.C. (1937). Variable Frequency Electric Circuit Theory with Application to the Theory of Frequency-Modulation. Bell Syst. Tech. J..

[7] Van der Pol B. (1946). The fundamental principles of frequency modulation. J. Inst. Electr. Eng.-Part III Radio Commun. Eng..

[8] Gabor D. (1946). Theory of communication. Part 1: The analysis of information. Electr. Eng. Part III Radio Commun. Eng..

[9] Ville J. (1948). Theorie et application dela notion de signal analysis. Cables Transm..

[10] Bedrosian E. (1963). A product theorem for Hilbert transforms. Proc. IEEE.

[11] Boashash B. (1992). Estimating and interpreting the instantaneous frequency of a signal. I. Fundamentals. Proc. IEEE.

[12] Qian T., Zhang L., Li H. (2008). Mono-components vs IMFs in signal decomposition. Int. J. Wavelets Multiresolution Inf. Process..

[13] Qian T. (2006). Mono-components for decomposition of signals. Math. Methods Appl. Sci..

[14] Wang Z., Wong C.M., Rosa A., Qian T., Wan F. (2022). Adaptive Fourier Decomposition for Multi-Channel Signal Analysis. IEEE Trans. Signal Process..

[15] Sharpley R.C., Vatchev V. (2006). Analysis of the Intrinsic Mode Functions. Constr. Approx..

[16] Huang N.E., Shen Z., Long S.R., Wu M.C., Shih H.H., Zheng Q., Yen N.-C., Tung C.C., Liu H.H. (1998). The empirical mode decomposition and the Hilbert spectrum for nonlinear and non-stationary time series analysis. Proc. R. Soc. Lond. Ser. A Math. Phys. Eng. Sci..

[17] Tian Y., Liu M., Zhang S., Zhou T. (2022). Underwater multi-target passive detection based on transient signals using adaptive empirical mode decomposition. Appl. Acoust..

[18] Wu Z., Huang N.E. (2009). Ensemble empirical mode decomposition: A noise-assisted data analysis method. Adv. Adapt. Data Anal..

[19] Dragomiretskiy K., Zosso D. (2014). Variational Mode Decomposition. IEEE Trans. Signal Process..

[20] Lei W., Wang G., Wan B., Min Y., Wu J., Li B. (2024). High voltage shunt reactor acoustic signal denoising based on the combination of VMD parameters optimized by coati optimization algorithm and wavelet threshold. Measurement.

[21] Tang L., Shang X.-Q., Huang T.-L., Wang N.-B., Ren W.-X. (2023). An improved local maximum synchrosqueezing transform with adaptive window width for instantaneous frequency identification of time-varying structures. Eng. Struct..

[22] Tian Y., Zhang J. (2020). Structural flexibility identification via moving-vehicle-induced time-varying modal parameters. J. Sound Vib..

[23] Ni P., Li J., Hao H., Xia Y., Wang X., Lee J.-M., Jung K.-H. (2018). Time-varying system identification using variational mode decomposition. Struct. Control Health Monit..

[24] Qian T. (2014). Adaptive Fourier decompositions and rational approximations, part I: Theory. Int. J. Wavelets Multiresolution Inf. Process..

[25] Qian T., Zhang L., Li Z. (2011). Algorithm of Adaptive Fourier Decomposition. IEEE Trans. Signal Process..

[26] Hou T.Y., Shi Z. (2011). Adaptive data analysis via sparse time-frequency representation. Adv. Adapt. Data Anal..

[27] Hou T.Y., Yan M.P., Wu Z. (2009). A variant of the EMD method for multi-scale data. Adv. Adapt. Data Anal..

[28] Wang Y., Markert R. (2016). Filter bank property of variational mode decomposition and its applications. Signal Process..

[29] Daubechies I., Lu J., Wu H.-T. (2011). Synchrosqueezed wavelet transforms: An empirical mode decomposition-like tool. Appl. Comput. Harmon. Anal..

[30] Gilles J. (2013). Empirical Wavelet Transform. IEEE Trans. Signal Process..

[31] Zhou F., Cicone A., Zhou H.M. (2024). IRCNN: A novel signal decomposition approach based on iterative residue convolutional neural network. Pattern Recognit..

[32] Zhao D., Shao D., Cui L. (2024). CTNet: A data-driven time-frequency technique for wind turbines fault diagnosis under time-varying speeds. ISA Trans..

[33] Dougherty R.L., Edelman A.S., Hyman J.M. (1989). Nonnegativity-, monotonicity-, or convexity-preserving cubic and quintic Hermite interpolation. Math. Comput..

[34] Fritsch F.N., Carlson R.E. (1980). Monotone Piecewise Cubic Interpolation. SIAM J. Numer. Anal..

[35] Ferguson J., Miller K. (1969). Characterization of shape in a class of third degree algebraic curves. TRW Rep..

